# Synthesis, Physicochemical Characterization and Applications of Advanced Nanomaterials

**DOI:** 10.3390/ma16041674

**Published:** 2023-02-17

**Authors:** Thomas Dippong

**Affiliations:** Faculty of Science, Technical University of Cluj-Napoca, 76 Victoriei Street, 430122 Baia Mare, Romania; dippong.thomas@yahoo.ro

**Keywords:** advanced metal oxide nanoparticles, synthesis, photocatalysts, sustainable processes, energy conversion, nanosensors, smart nanostructures and nanodevices for virus detection

## Abstract

This Special Issue highlights the last decade’s progress regarding new nanostructured materials. In this regard, the development of nanoscale syntheses and innovative characterization tools that resulted in the tailored design of nanostructured materials with versatile abilities in many applications were investigated. Various types of engineered nanostructures, usually metal nanoparticles or nanoporous metal oxides, have been synthesized for various applications. This Special Issue covers the state-of-the-art of advanced nanoparticles in many disciplines (chemistry, pharmacy, nanomedicine, agriculture, catalysis, and environmental science). The crystallite sizes depended on the annealing temperature and type of doping ion. A combination of rigid and soft particles could simultaneously enhance both the tensile properties and the fracture toughness, which could not be achieved by the single-phase particles independently. The surface charge and in vitro corrosion resistance are key parameters characterizing biomaterials in the interaction of the implant with the biological environment. Solar energy in the presence of a photocatalyst can be effectively converted into electricity/fuel, break down chemical and microbial pollutants, and help water purification. The saturation magnetization, remanent magnetizations, coercivity, and anisotropy were found to depend on the doping ion, annealing temperature, and particle size. The efficiency of the photocatalysis reaction depends on several factors, including light absorption capacity/light intensity, the type of photocatalyst used, the concentration of a photocatalyst and contaminant particles, the pH of the reaction medium, etc. The variety of color pigments and coloring properties of the targeted application in the ceramic industry was also of interest.

With the rapid development of nanotechnology, nanomaterials have recently attracted the attention of the scientific community due to their unique structural, morphological, optical, electrical, thermal, and magnetic characteristics [1,2,3]. These enhanced properties are caused by their high surface-to-volume ratio due to their size falling in the 1–100 nm range [1,2,3]. Nanomaterials can be metallic-based nanoparticles (ferrites, chromates, aluminates, bismutates, etc.) or carbon oxides (carbon nanotubes, graphenes, graphene oxides, etc.). The tailoring of the shape, size, and size distribution of nanoparticles, and the properties of hybrid nanoparticles, is achieved through different synthesis routes by modifying parameters such as the pH, concentration of reactants, dopants, or stirring speed. Some of these methods are complex, involving the use of reduction agents with little to no impact on the environment and needing a longer reaction time or a high processing temperature to complete crystallization [1,2,3]. These tailored properties of nanoparticles make them suitable candidates for technological applications in photocatalysis, photoluminescence, biosensors, catalysis, humidity sensors, permanent magnets, magnetic drug delivery, magnetic liquids, magnetic refrigeration, ceramic pigments, microwave absorbents, corrosion protection, water decontamination, photocatalysis, antimicrobial agents or biomedicine (hyperthermia) [1,2,3]. Multifunctional magnetic nanocomposites are among those heterogeneous nanosized systems where at least one phase component is magnetic and can act as an intermediate of either the actuation or the response of the overall system. The main advantage of heterogeneous nanosystems is the possibility of combining and inter-influencing the electronic properties of the constituent interfaced nanophases.

The Special Issue, entitled *Synthesis, Characterization and Application of Sustainable Advanced Nanomaterials*, includes 18 original research works and focuses on highlighting the progress, challenges, and future directions in the area of the synthesis and characterization of nanomaterials and nanostructures with multiple applications in chemistry, physics, biology, and medicine [1,2,3]. 

A sol–gel route followed by thermal treatment used to produce NiFe_2_O_4_ doped with transition metal ions (Zn^2+^, Mn^2+^, Co^2+^) was reported by Dippong et al. [4]. The TG/DTA curves of samples dried at 40 °C indicated the formation and decomposition of metallic precursors to ferrites in single or two stages, with comparable mass losses [4]. The functional groups identified by Fourier-transform infrared spectroscopy confirmed the decomposition of metal nitrates, the formation and decomposition of precursors, and the formation of the SiO_2_ matrix [4]. The X-ray diffraction indicated that the sol–gel synthesis produced single-phase crystalline ferrites in the case of the Zn^2+^- and Co^2+^-doped Ni-ferrites. By doping with Mn^2+^, several secondary phases derived from the SiO_2_ matrix (cristobalite, quartz, and Fe_2_SiO_4_) accompanied the crystalline spinel ferrite. The XRD parameters were influenced by the crystallite size, lattice strain, defects, annealing temperature, and doping ions. The gradual increase of the lattice parameters suggested the uniform distribution of doping metal ions in the NiFe_2_O_4_ lattice. The unit cell volume increases by doping with Mn^2+^ ion and decreases by doping with Zn^2+^ and Co^2+^ ions. By contrast, the X-ray and bulk densities and porosity decrease by doping with Mn^2+^ and increase with doping Zn^2+^ and Co^2+^ ions [4]. The NiFe_2_O_4_ particle size increases by doping with Mn^2+^ and decreases by doping with Zn^2+^ and Co^2+^ ions, respectively. The doping of NiFe_2_O_4_ with Zn^2+^, Mn^2+^, and Co^2+^ leads to a decrease of the saturation magnetization and remanent magnetization, whereas the coercivity decreases at 700 °C and increases at 1000 °C. The obtained magnetic transition metal dopped-Ni ferrite nanoparticles are possible candidates for various medical applications such as controlled drug delivery, cancer therapy, biosensing, and magnetic resonance imaging [4].

In this Special Issue, Goga et al. [5], focused on the Ni substitution of cobalt ions in a Co_x_Cr_2_O_4_ matrix (x = 0, 0.25, 0.5, 0.75, and 1.00) by using a sol–gel synthesis route, was performed. The X-ray diffraction (XRD) studies reveal a spinel-type Face–Centered Cubic structure and a secondary Cr_2_O_3_ phase when x ≤ 0.75 and a Body–Centered Tetragonal structure when x = 1. The structural characterization is consistent with the Ni^2+^ substitution of Co^2+^ ions, thereby a decrease of unit cell parameter and the unit cell volume was observed with an increase of x. The increase of Ni^2+^ substitution in the matrix has a pronounced increase in the size of the crystallites from 39.9 nm (for x = 0) to 99.42 nm (for x = 0.75) [5]. FT–IR indicated two strong absorption bands corresponding to the metal–oxygen stretching from tetrahedral and octahedral sites, characteristic of a spinel structure. The UV-VIS absorption bands assigned to the Co_(1−x)_Ni_x_Cr_2_O_4_ spinel confirm the Ni^2+^ ions at the A site and the Cr^3+^ ions at the B sites. Adjusting the nickel content in the CoCr_2_O_4_ matrix, the color of the pigment can be easily controlled. Lighter shades of ceramic glazes can be obtained when embedding in glossy glaze and darker shades can be obtained when embedding in a matte glaze, confirming the spinels’ applicability as pigments in the ceramic tile industry [5]. SEM and TEM microscopy evidenced the powder morphology and the tendency of nanoparticles to agglomerate. The elemental EDX distribution of Ni Kα1 in the glossy ceramic tile confirms the homogeneous and uniform pigment distribution in the glaze after firing. All the samples present distinct stable colors and good structural and morphological properties that recommend them to be used as ceramic pigments [5].

In this Special Issue, Feliczak-Guzik [6] reviewed nanomaterials as photocatalysts—synthesis and their potential applications. The increasing demand for energy and environmental degradation are current serious problems. Therefore, searching for new, efficient, and stable photocatalysts with high application potential is a point of great interest. Over the years, research on the synthesis of photocatalysts has evolved considerably from transition metal oxides (e.g., TiO_2_/ZnO) to much more advanced materials [6]. The photocatalysts should be characterized by the ability to absorb radiation from a wide spectral range of light, the appropriate position of the semiconductor energy bands to the redox reaction potentials, and the long diffusion path of charge carriers, besides the thermodynamic, electrochemical, and photoelectrochemical stabilities. The light absorption capacity/light intensity, the type of photocatalyst used, the concentration of a photocatalyst and contaminant particles, and reaction medium’s pH are key factors to determining the optimal amount of these factors for a given photocatalyst and type of pollutant [6]. Therefore, efforts are being performed to increase the efficiency of photo processes by changing the electron structure, surface morphology, and crystal structure of semiconductors. The photocatalysis process has been studied for a long time on a laboratory scale; however, its large-scale application is greatly hampered (by blocking light penetration in thick coatings, leaching effects, and difficulty in recovering the photocatalyst). In natural systems, the decomposition rate of pollutants is not limited by a time regime, unlike in industrial installations, where the technical challenge of photocatalytic processes, especially in heterogeneous systems, is unsatisfactory reaction kinetics [6].

Oumezzine et al. [7] reported the magnetocaloric and giant magnetoresistance effects in La-Ba-Mn-Ti-O epitaxial thin films: the influence of phase transition and magnetic anisotropy. Magnetic perovskite films have promising properties for energy-efficient spintronic devices and magnetic refrigeration. Here, an epitaxial ferromagnetic La_0.67_Ba_0.33_Mn_0.95_Ti_0.05_O_3_ thin film was grown on a SrTiO_3_ single crystal substrate by pulsed laser deposition. High-resolution X-ray diffraction proved the high crystallinity of the film with tetragonal symmetry. The magnetic, magnetocaloric effect and magnetoresistance of the La_0.67_Ba_0.33_Mn_0.95_Ti_0.05_O_3_ film with a thickness of 97 nm have been studied at different directions of the applied magnetic field to the sample plane. The La_0.67_Ba_0.33_Mn_0.95_Ti_0.05_O_3_ epilayer exhibits a second-order ferromagnetic phase transition around 234 K together with a metal–semiconductor transition close to this Curie temperature (T_C_) and an in-plane magnetic uniaxial easy axis. Further, a gradual metal-to-paramagnetic semiconductor transition at higher temperature finishes at 245 K. The magnetic entropy variation under 5 T induction of a magnetic field applied parallel to the film surface reaches a maximum of 17.27 mJ/cm^3^ K, and the relative cooling power is 1400 mJ/cm^3^ K for the same applied magnetic field [7]. Another important finding is that the La_0.67_Ba_0.33_Mn_0.95_Ti_0.05_O_3_ epitaxial thin film has a giant magnetoresistance as high as 82% at a temperature close to the T_C_, which may be interesting for electromagnetic applications. 

In this Special Issue, Lenar et al. [8] present a new reliable pH sensor based on hydrous iridium dioxide and its composites. The addition of a conducting polymer to the composite material changed the wetting properties of the material, making it highly hydrophobic, which consequently contributed to the stability of the potentiometric response during the water-layer test. Three hIrO_2_-based materials were prepared and applied as solid-contact layers in pH-selective electrodes with polymeric membranes [8]. The material included a standalone hydrous iridium oxide; a composite material of hydrous iridium oxide, carbon nanotubes, and triple composite material composed of hydrous iridium oxide; carbon nanotubes; and poly(3-octylthiophene-2,5-diyl) [8]. Each component contributed differently to the sensors’ performance—the addition of carbon nanotubes increased the electrical capacitance of the sensor. Oppositely, the addition of the conducting polymer allowed it to increase the contact angle of the material, changing its wetting properties and enhancing the stability of the potentiometric response. The hydrous iridium oxide-contacted electrodes exhibit linear responses in a wide linear range of pH (2–11) and stable potentiometric responses (the lowest potential drift of 0.036 mV/h is attributed to the electrode with triple composite material). The response towards hydrogen ions turned out to be repeatable and reversible within this range of pH values, and neither redox nor light sensitivity were detected. No presence of a water layer was detected in the solid-contact electrodes with IrO_2_^−^-based materials [8].

The study of Hsu et al. [9], focusing on the modification of electrospun CeO_2_ nanofibers with CuCrO_2_ particles applied to hydrogen harvest from steam reforming of methanol, was also included in this Special Issue. Hydrogen is an alternative renewable energy source for addressing the energy crisis and climate change. CuCrO_2_ particles were attached to the surfaces of electrospun CeO_2_ nanofibers to form CeO_2_-CuCrO_2_ nanofibers; the catalyst was produced and used for steam reforming of methanol. The CuCrO_2_ particles did not readily adhere to the surfaces of the CeO_2_ nanofibers, so a trace amount of SiO_2_ was added to the surfaces to make them hydrophilic. After the SiO_2_ modification, the CeO_2_ nanofibers were immersed in a Cu-Cr-O precursor and annealed in a vacuum to form CeO_2_-CuCrO_2_ nanofibers. The specific surface area of the CeO_2_-CuCrO_2_ nanofibers is 15.06 m^2^/g [9]. According to the findings, the increased hydrogen production rate can be ascribed to the stronger catalytic activity, larger surface area, lower reactor temperature, and higher methanol flow rate of the CeO_2_-CuCrO_2_ nanofiber catalyst. According to the H_2_ production performance, the CeO_2_-CuCrO_2_ nanofibers can be a better catalyst for commercial H_2_ production and are suitable for fuel cell vehicles without high-temperature activation [9].

The study of Atanasov et al. [10] compares the structural and magnetic properties of the nano- and polycrystalline manganites La_(0.7−x)_Eu_x_Ba_0.3_MnO_3_, which are potential magnetocaloric materials to be used in domestic magnetic refrigeration close to room temperature. The sol–gel method produced nano-scale particles, showing an average size of 30–70 nm. Both systems are single-phase, with rhombohedral lattice symmetry. Iodometry was used to estimate the oxygen content in samples, showing a lower concentration of Mn^4+^ ions, leading to the lowest oxygen content value of O_2.97±0.02_ for the x = 0.4 sample. To reduce this temperature below 300 K, the La^3+^ ions were partially replaced by Eu ions. In nano-sized manganites, the reduction of T_C_ is accompanied by a broad magnetic transition, extending the magnetic cooling effect to a larger temperature range [10]. The magnetic measurements revealed single magnetic phases, low magnetic anisotropy, and very small coercivity for both systems. The bulk samples with x < 0.4 show a metallic–insulator transition at a temperature *T*_p_ lower than magnetic transition temperature *T*_c_. All samples show a negative magnetoresistance. A modified Arrott plot analysis revealed that bulk samples’ critical exponents were in the tricritical mean field model range and in the 3D Heisenberg model range for nanocrystalline samples. The maximum magnetic entropy change of 4.2 J/kgK was observed for the x = 0.05 bulk sample for µ_0_Δ*H* = 4 T [10]. Since the temperature range (δ*T_FWHM_*) for nano-sized samples La_0.7_Ba_0.3_MnO_3_ and La_0.65_Eu_0.05_Ba_0.3_MnO_3_ covers a wide range including room temperature, they may be used in multistep refrigeration processes. The magnetocaloric effect was found to be larger and close to room temperature for the bulk samples, while for the nano-samples, it was lower, but extended on a large temperature range. This wide range of effective nanoparticle cooling and high entropy change in bulk material can be combined for suitable commercial cooling [10].

Cepoi et al. [11] presented that *Arthrospira platensis* easily tolerates the presence of high concentrations of selenium (up to 125 mg/L) in the medium, growth, and biomass accumulation, being within the limits of the values characteristic for the control biomass. The biosynthesis of selenium nanoparticles has become particularly important due to the environmentally friendly character of the process and the special properties of the obtained particles. For selenium concentrations up to 50 mg/L, the amount of biomass accumulated during the cultivation cycle increased by up to 18% compared to the control [11]. The content of lipids and carbohydrates in biomass increased with the increasing sodium selenite concentration added to the nutrient medium. The content of protein and phycobilin also increased, and the dose-dependent character of this relationship was maintained up to a concentration of sodium selenite of 175 mg/L [11]. With an increase in the content of lipids, the level of malonic dialdehyde in the cells also increased. Most of the bioaccumulated selenium was determined in the protein (47.5% of the accumulated selenium) and the lipid (24.1%) fractions, the ultrastructural changes in the cells during biosynthesis and the change in the expression of some genes involved in stress response reactions [11]. In the protein fraction, selenium nanoparticles with a size of 2–8 nm were formed. Thus, the expression level of iron-superoxide dismutase and heat-shock protein increased, which may be associated with the need to manage the increased flow of reactive oxygen species and to stabilize the proteins subjected to the action of the xenobiotics. Selenium also caused ultrastructural changes in *Arthrospira platensis* expressed in the damage and disorder of thylakoids, the detachment of the cytoplasmic membrane from the cell wall, the change in the density of the cell wall, and the formation of carbon reserves in the cells, indicating the negative effects of selenium ions [11]. Thus, *Arthrospira platensis* tolerates high concentrations of selenium, accumulates significant amounts of this element, and carries out the biosynthesis of selenium nanoparticles, which are mainly located in the protein and lipid fractions. The process is accompanied by biochemical, ultrastructural, and gene expression changes associated with the response of spirulina to stress conditions [11].

Glaskova-Kuzmina et al. [12] reported on the effect of core–shell rubber nanoparticles on the tensile properties, fracture toughness, and glass transition temperature of the epoxy and epoxy-based carbon fiber reinforced polymer. The Hansen model was applied to describe the elastic modulus of the epoxy possessing a certain fraction of the core–shell rubber nanoparticles and pores. Three additives containing core–shell rubber nanoparticles were used for the research, resulting in a filler fraction of 2–6 wt.% in the epoxy resin. The effect of the core–shell rubber nanoparticles on the tensile properties of the epoxy resin was notable, leading to a reduction of 10–20% in the tensile strength and elastic modulus and an increase of 60–108% in the fracture toughness for the highest filler fraction. No considerable distinction in the fracture toughness among the additives was detected, thereby proving that the small (100 nm) and large (300 nm) core–shell rubber nanoparticles were equally efficient [12]. The glass transition temperature of the epoxy was gradually improved by 10–20 °C with the increase of core–shell rubber nanoparticles for all of the additives, which could be attributed to the high crosslink density and toughening effect of rubber modifiers, thereby testifying to their dissolution in the continuous epoxy phase. The possible combination of rigid and soft particles could be a compromise to simultaneously improve both the tensile properties and the fracture toughness, which cannot be achieved by the single-phase particles independently [12].

Che et al. [13] presented a novel dual-emission fluorescence probe based on carbon dots and an Eu^3+^ functionalized UiO-66-(COOH)_2_ hybrid for visual monitoring of Cu^2+^. The carbon dots-UiO-66-(COOH)_2_ exhibits outstanding selectivity, excellent sensitivity, and good anti-interference for ratiometric sensing Cu^2+^ in water. The linear range is 0–200 μM, and the detection limit is 0.409 μM [13]. The carbon dots-UiO-66-(COOH)_2_ silicon plate achieves rapid and selective detection of Cu^2+^ and the change in fluorescence color can be observed by the naked eye [13]. These results reveal that the carbon dots-UiO-66-(COOH)_2_ hybrid can be employed as a simple, rapid, and sensitive fluorescent probe to detect Cu^2+^ [13]. The possible sensing mechanism of this dual-emission fluorescent probe is discussed in detail [13]. The result reveals that adding Cu^2+^ would affect the energy transfer between the ligand and Eu^3+^, which would quench the luminescence of Eu^3+^. This finding indicates that carbon dots-UiO-66-(COOH)_2_ material can be employed as a fluorescent probe to rapidly and efficiently detect Cu^2+^ in aqueous solutions [13]. 

Krajczewski et al. [14] reported on the WO_3_ nanopores array modified by Au trisoctahedral NPs: formation, characterization and SERS application. The WO_3_ nanopores array was obtained by an anodization method in an aqueous solution with the addition of F^-^ ions. Several factors affecting the final morphology of the samples were tested, such as potential, time, and F^-^ concentrations. Using smaller trisoctahedron Au NPs as seeds for the growth of larger nanoparticles permits easy tuning of the size of particles while maintaining the well-defined trisoctahedron shape. The nanopore’s size increased with the increasing potential [14]. The XPS measurements do not show any contamination by F^-^ on the surface, typical for WO_x_ samples formed by an anodization method in the range of 0.5–1 h. Such a layer was successfully modified by anisotropic gold trisoctahedral NPs of various sizes. The UV-Vis spectroscopy showed shifting of SPR into longer wavelengths with the successive growth of nanoparticles. The WO_3_—Au trisoctahedron-modified nanoarray was successfully used as a SERS platform. The highest enhancement was observed for the Au NPs with a 94 nm diameter [14].

Saha et al. [15] effectively synthesized sulfur-doped nanoporous carbon with an ultrahigh surface area from lignin by one-step carbonization with the help of sodium thiosulfate as a sulfurizing agent and potassium hydroxide as an activating agent to create porosity. Lignin is the second-most available biopolymer in nature. Lignin was employed as the carbon precursor for the one-step synthesis of sulfur-doped nanoporous carbons and has several applications in scientific and technological sectors. The peak deconvolution results of XPS confirmed that the nanoporous carbons possess sulfur contents of 1 to 12.6 at.%, and the key functionalities include S=C, S-C=O, and SO_x_. The nanoporous carbons’ porosity analysis revealed that the BET-specific surface areas of the carbons are in 741–3626 m^2^/g and a total pore volume of 0.5–1.74 cm^3^/g [15]. The surface area of 3626 m^2^/g is one of the highest for carbon-based materials reported in the literature [15]. Pure-component adsorption isotherms of CO_2_, CH_4_, and N_2_ were measured on all the porous carbons at 298 K, with a pressure up to 760 torrs [15]. The carbon with the highest BET surface area demonstrated the highest CO_2_ uptake of more than 10.89 mmol/g, at 298 K and 760 torr, which is one of the highest for porous carbon-based materials, compared to previous studies [15]. Ideally, the adsorbed solution theory was employed to calculate the selectivity for CO_2_/N_2_, CO_2_/CH_4_, and CH_4_/N_2_, from the pure-component isotherm data, and some of the carbons reported a very high selectivity value. 

Petean et al. [16] presented the silver depreciation in 3-Polker coins issued from 1619–1627 by Sigismund III Vasa, King of Poland, in the context of the “Kipper- und Wipperzeit” financial crisis generated by the 30-years war, using non-destructive investigation methods such as X-ray diffraction and Scanning Electron Microscopy coupled with Energy Dispersion Spectroscopy (EDS) elemental analysis. It was characterized by a strong debasing of the silver title of the coins issued by the countries involved in the war. Silver coins issued by Poland were generally considered safer. Some historical references mention forgeries of this monetary type issued in copper plated with a thin silver foil. Using modern material investigation techniques, the authors aimed to find the precise situation of the officially issued 3-Polker by the Poland mints. A significant achievement of this research is the SEM–EDS elemental maps recorded for each coin that reveal the silver alpha phase grains and Ag-Cu eutectic grains without metallographic analysis [16]. These methods allow proper investigation of the coins and preserve their integrity, a necessary factor for valuable museum artifacts. The findings reveal important facts for historians: the 3-Polker coins issued by Sigismund III Vasa, King of Poland, from 1619–1627 evidenced a certain depreciation of the silver title from about 84.3% to a range of 63.2–74.6% for the coins issued between 1621–1625 [16]. It is a mild decrease in the silver title compared to the historical data regarding the currency affected by the Kipper- und Wipperzeit crisis. The findings reveal that the silver title in 3-Polker coins was restored to the normal value between 1626 and 1627. The author concludes that the 3-Polker issued in the official Poland mints, even those affected by silver depreciation, was considered good money (being hoarded) and definitely could not be the rich copper debased coins mentioned in some of the medieval sources [16].

Iosif et al. [17] reported on the mechanical properties of orthodontic cements and their behavior in acidic environments and investigated the mechanical properties and morphology of three categories of orthodontic cements: resin composites (BracePaste); resin-modified glass ionomer (Fuji Ortho) and resin cement (Transbond) exposed to acidic environments such as Coca Cola^TM^ and Red Bull^TM^. Their mechanical properties, such as compressive strength, diametral tensile strength, and flexural strength, were correlated with the samples’ microstructures, liquid absorption, and solubility in liquid [17]. The findings suggest that Transbond resin cement presents the best compression strength and BracePaste features the best flexural strength. The elastic modulus is very important considering the solicitations induced by chewing forces. The BracePaste has the best value of the elastic modulus, followed by Fuji Ortho. Therefore, each material has strong points that are useful for personalized orthodontic treatment according to the patient’s requirements. Acid soft drinks and energy drinks are very popular among young patients with orthodontic brackets. The acidic components within these soft drinks (phosphoric acid in Coca-Cola^TM^ and citric acid in Red Bull^TM^) can erode the bonding layer and affect the bracket’s stability. Atomic force microscopy reveals the nanostructural alteration of the investigated orthodontic materials, such as roughness increasing and nano-filler particles acid erosion [17]. It was found that these parameters strongly influence the orthodontic material behavior (e.g., BracePaste roughness decreasing under acid exposure proves an excellent resistance to in-depth erosive penetration), a fact that must be considered when the orthodontic treatment is prescribed to the patient. BracePaste is recommended for long-term orthodontic treatment for patients who regularly consume acidic beverages, Fuji Ortho is recommended for short-term orthodontic treatment for patients who regularly consume acidic beverages, and Transbond is recommended for orthodontic treatment over an average time period for patients who do not regularly consume acidic beverages.

The study by Turza et al. [18] showed the structural aspects and intermolecular energy of some short testosterone esters. Testosterone (17β-Hydroxyandrost-4-en-3-one) is the primary male anabolic–androgenic steroid. A single crystal X-ray diffraction technique was employed to elucidate the crystal structures of three short testosterone esters: propionate, phenylpropionate, and isocaproate. They were shown to belong to the non-centrosymmetric orthorhombic P2_1_2_1_2_1_, and monoclinic P2_1_ space groups. Structural features were described and evaluated in terms of Hirshfeld surfaces, and crystal energies were further compared with the base native form (without ester) and with the acetate ester [18]. The investigation of crystals in the solid state via computational methods yielded that, in all crystals, the crystal stability and formation of supramolecular self-assemblies are governed by dominant dispersion effects. Although the C-H-O hydrogen bonds are present in all compounds, they play a less noticeable role [18]. Total crystal lattice energies are greater in absolute terms with the increase in ester chain length. The core steroidal rings depict similar conformations in all prodrugs, with the six-membered A rings in intermediate sofa-half-chair geometries, B and C rings showing chair-like conformations, and five-membered D rings showing intermediate envelope-half-chair conformations. The molecular overlap indicates a good match of backbone skeleton rings representing the native part of the ester’s structures and the differences occurring in the carbon tails orientation. From a pharmaceutical point of view, their solubility is correlated with ester length, which implies the added ester functionalities. The shortest acetate ester possesses the lowest solubility, while the longest isocaproate ester is approximately four-fold greater. Phenylpropionate and propionate forms show similar values and are between the other two [18].

The influence of anodizing conditions on the biotribological and micromechanical properties of Ti–13Zr–13Nb alloy was reported by Stróz et al. [19]. The porous oxide nanotubes’ layers of various geometries and lengths on the Ti–13Zr–13Nb alloy surface can be produced by anodizing to improve osseointegration, which shows that Vickers microhardness determined under variable loads changed depending on the type of electrolyte and applied voltage–time parameters of electrochemical oxidation. By anodizing, first-generation, second-generation, and third-generation oxide nanotubes layers were produced on the Ti–13Zr–13Nb alloy surface. Vickers microhardness decreased from 181(5) to 252(6) and from 254(3) to 221(3) with the increasing load for second-generation and third-generation oxide nanotube layers, respectively, compared to the alloy substrate [19]. The kinetic coefficient of friction determined based on the friction coefficient took the smallest value of 0.86(8) for the second-generation oxide nanotubes’ layer. The highest coefficient of kinetic friction of 0.94(1) was characterized by the surface of the first-generation oxide nanotubes’ layer [19]. Based on the results obtained, a three-body abrasion wear mechanism was proposed for biotribological wear of the Ti–13Zr–13Nb alloy before and after anodizing in Ringer’s solution. Based on the biotribological tests carried out in Ringer’s solution in a reciprocating motion in the ball-on-flat system for the Ti–13Nb–13Zr alloy before and after anodizing, it was found that the non-anodized alloy was characterized by the highest wear resistance for which the average material volume consumption. Wear scars’ analysis of the ZrO_2_ ball was performed using optical microscopy. It was found that the composition of the electrolyte with the presence of fluoride ions was an essential factor influencing the micromechanical and biotribological properties of the obtained oxide nanotubes’ layers [19]. 

Stróż et al. [20] reported in vitro bioelectrochemical properties of second-generation oxide nanotubes on a Ti–13Zr–13Nb biomedical alloy. In a neutral aqueous KCl solution, the second-generation oxide nanotubes layer moves the isoelectric point from 4.2 for the non-anodized Ti–13Zr–13Nb alloy, which is typical for the surface without a functional group to pH of 5.4, which is characteristic for the amorphous oxide phase. Comparison of the influence of different electrolytes such as KCl, PBS, and artificial blood on the zeta potential at pH of 7.4 for the Ti–13Zr–13Nb alloy before and after anodizing revealed a strong reaction of calcium anions with amorphous surfaces [20]. The complex ions in artificial blood have demonstrated a stronger affinity to the hydrophobic surface before anodizing than hydrophilic ones after electrochemical oxidation. The increase in the corrosion resistance of the anodized Ti–13Zr–13Nb electrode in PBS compared with the non-anodized Ti–13Zr–13Nb electrode was due to the presence of a stable second-generation oxide nanotubes layer. The zeta potential method used in these in vitro studies could not be used in vivo due to technical limitations, determination of the breakdown potential of the second-generation oxide nanotubes layer on the Ti–13Zr–13Nb alloy in PBS was not possible due to the technical limitations of the potentiostat to the tested potential range of 10 V. Knowledge about the kinetics of drug release from the obtained oxide nanotubes will facilitate the development of personalized implants that are carriers of tissue-forming and therapeutic substances, supporting the process of osseointegration of the implant in the human body [20].

In the last Special Issue article, Valh [21] investigated extending the protection ability and life cycle of medical masks through the washing process. Numerous challenges and the pandemic period of SARS-CoV-2 affecting people’s respiratory systems have raised specific questions and doubts about the extent to which consumer laundry detergents can reasonably ensure the level of disinfection during washing. Reusing decontaminated disposable medical face masks could contribute to reducing the environmental burden of discarded masks. The hydrophobicity of medical masks determined through the static contact angle depends on the number of cycles carried out. The static contact angle of the samples after the first cycle is lower than after the fifth cycle in all procedures. The barrier properties of the medical mask were analyzed before and after the first and fifth washing cycles indirectly by measuring the contact angle of the liquid droplets with the front and back surface of the mask and further by measuring the air permeability and determining the antimicrobial resistance. Images of ultrapure water drops on the surface confirm the hydrophobicity of the front/back of the medical mask before and after washing. The additional analysis included FT-IR, pH of the material surface and aqueous extract, and the determination of residual substances—surfactants—in the aqueous extract of washed versus unwashed medical masks, while their aesthetic aspect was examined by measuring their spectral characteristics [21,22]. The results showed that household washing had a more substantial impact on the change of some functional properties, primarily air permeability, than laboratory washing [22]. The disinfectant agent, didecyldimethylammonium chloride, contributes to the protective ability and supports the idea that washing medical masks under controlled conditions can preserve barrier properties and enable reusability [21].

I am aware that the diversity and innovation of new compounds and tools rapidly developing in multidisciplinary research related to nanomaterials based on metals cannot all be collected in a single volume. However, this collection will contribute to the interest of research in this area, providing our readers with a broad and updated scenario. All these published studies will offer a new approach for future studies to create important advances in materials science and engineering.

In conclusion, as the Editors of this Special Issue, we would like to thank all the authors and reviewers who contributed to this Special Issue with innovative ideas and constructive reviewers’ comments. We are grateful for the consistent support from the *Materials* Editorial Office. We are sure that this Special Issue will provide our readers with a platform to understand the novel real-world synthesis and characterization of innovative nanomaterials and nanostructures with their pivotal roles in diverse applications.
